# Recurrent Vasovagal Syncope in a Patient Receiving Pembrolizumab for Postoperative Pulmonary Metastases of Laryngeal Cancer: A Case Report

**DOI:** 10.7759/cureus.95330

**Published:** 2025-10-24

**Authors:** Rentaro Takeuchi, Kunihiko Tokashiki, Yasuo Ogawa, Shun Mochida, Kiyoaki Tsukahara

**Affiliations:** 1 Otolaryngology-Head and Neck Surgery, Tokyo Medical University, Tokyo, JPN; 2 Otolaryngology-Head and Neck Surgery, Tokyo Medical University Hachioji Medical Center, Tokyo, JPN

**Keywords:** immune checkpoint inhibitor, immune-related adverse event, laryngeal cancer, pembrolizumab, pulmonary metastases, vasovagal syncope

## Abstract

Immune checkpoint inhibitors (ICIs), such as pembrolizumab, are widely used for the treatment of various malignancies. Immune-related adverse events (irAEs), including neurological, endocrine, and cardiovascular complications, are known side effects of ICIs. Although syncope can occur as a manifestation of irAEs, vasovagal syncope (VVS), one of the most common causes of transient loss of consciousness, is not typically considered an irAE. Herein, we report a case of a 72-year-old man with postoperative pulmonary metastases from laryngeal cancer who underwent long-term pembrolizumab monotherapy. After completing 28 uneventful cycles, the patient experienced a transient episode of loss of consciousness immediately following the 29th infusion. The episode resolved spontaneously within a short period of time; however, the patient was admitted for evaluation. During this initial hospitalization, no further syncopal episodes occurred, and no remarkable abnormalities were found on cardiac, neurological, or endocrine assessments. The patient was discharged once his condition stabilized. However, shortly after discharge, he experienced a similar episode and was brought to our hospital. Upon arrival, his symptoms resolved, but he was readmitted for a second evaluation. During hospitalization, vasovagal syncope was diagnosed by the cardiology team based on head-up tilt testing and exclusion of other causes. The patient was managed using behavioral strategies and pharmacological therapy. Although pembrolizumab was temporarily suspended during the evaluation, it was resumed based on the confirmed diagnosis of VVS, absence of irAE-related findings, and a favorable oncologic response. This case emphasizes the importance of considering non-immune etiologies such as VVS in patients undergoing ICI therapy who present with transient loss of consciousness.

## Introduction

Immune checkpoint inhibitors (ICIs), such as pembrolizumab, an anti-PD-1 monoclonal antibody, have become a central component of the treatment for recurrent or metastatic head and neck cancers. Despite their clinical efficacy, ICIs are associated with a wide spectrum of immune-related adverse events (irAEs) that can affect various organ systems, including the gastrointestinal tract, endocrine glands, and nervous system, with an incidence rate of approximately 10% regardless of organ involvement or severity [1,2]. Although less frequent, neurological and cardiovascular manifestations can include severe complications, such as meningoradiculitis and myasthenia gravis [3]. Importantly, irAEs may present with non-specific symptoms, such as transient loss of consciousness, which require a broad differential diagnosis.

Vasovagal syncope (VVS), one of the most common causes of loss of consciousness, is typically caused by a transient autonomic imbalance in response to emotional or physical stimulation, including pain, micturition, defecation, or visual triggers, such as blood [4]. Although generally benign, VVS can severely impact the quality of life, particularly in elderly patients with recurrent episodes [5]. Diagnosis is often clinical; however, basic screening tests for autonomic nervous system function, such as the head-up tilt test, are occasionally performed. Although most cases are conservatively managed, pharmacological therapy or device-based interventions may be required for persistent or severe presentations. Here, we report a case of recurrent syncope during pembrolizumab therapy for postoperative pulmonary metastasis of laryngeal cancer. Although irAEs were initially considered, a thorough evaluation excluded immune-mediated causes, and the patient was ultimately diagnosed with vasovagal syncope.

## Case presentation

A 72-year-old man with stage IVA (pT4aN0M0) laryngeal cancer underwent total laryngectomy, bilateral neck dissection, total thyroidectomy, and pectoralis major flap reconstruction. Two years after surgery, imaging tests revealed new lung lesions, which were diagnosed as lung metastases, and he was initiated on pembrolizumab monotherapy.

**Figure 1:**
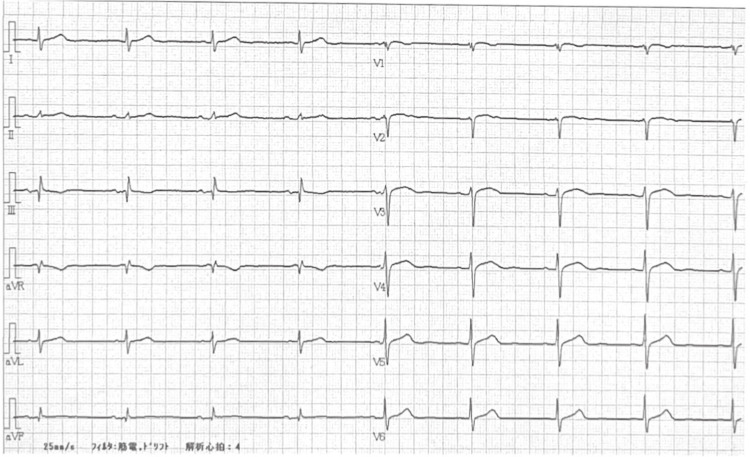
Electrocardiogram (ECG) at the first syncopal episode. A 12-lead ECG was recorded immediately after the first syncopal episode following the 29th pembrolizumab infusion. Sinus bradycardia (52 bpm) was observed without ST-T changes or arrhythmias.

After completing 28 uneventful cycles, the patient experienced a transient loss of consciousness immediately following the 29th infusion. These episodes resolved spontaneously within approximately 1 min. At the time of initial medical assessment, his vital signs were as follows: body temperature 36.1°C, heart rate 49 bpm, blood pressure 53/33 mmHg, respiratory rate 17 breaths/min, and SpO_2_ 99% on room air. Electrocardiogram (ECG) revealed a sinus rhythm at 52 bpm with no ST-T abnormalities (Figure 1). Physical examination revealed no accumulation of sputum or crusting within the permanent tracheostoma and no signs of airway obstruction. Blood tests performed before pembrolizumab administration revealed no significant abnormalities aside from known chronic kidney dysfunction (Table 1). Specifically, hematologic, hepatic, endocrine, and electrolyte parameters were within the normal ranges.

**Table 1:** Summary of the laboratory results. WBC: white blood cell; Hb: hemoglobin; AST: aspartate aminotransferase; ALT: alanine aminotransferase; BUN: blood urea nitrogen; eGFR: estimated glomerular filtration rate; Na: sodium; K: potassium; Cl: chloride; Ca: calcium; P: phosphorus; HbA1c: hemoglobin A1c; FT3: free triiodothyronine; FT4: free thyroxine; TSH: thyroid-stimulating hormone; ACTH: adrenocorticotropic hormone; KL-6: Krebs von den Lungen-6

Tests	Results	Reference range	Interpretation
WBC	5710/μL	4000-8000/μL	Normal
Hb	15.0 g/dL	13.5-17.5 g/dL	Normal
Platelets	23.6×10⁴/μL	15-35×10⁴/μL	Normal
Albumin	4.4 g/dL	4.1-5.1 g/dL	Normal
Total bilirubin	0.7 mg/dL	0-1.5 mg/dL	Normal
AST	15 IU/L	13-30 IU/L	Normal
ALT	12 IU/L	10-42 IU/L	Normal
BUN	18.8 mg/dL	8-20 mg/dL	Normal
Creatinine	1.1 mg/dL	0.65-1.07 mg/dL	Slightly elevated
eGFR	51.2 mL/min/1.73 m²	>60 mL/min/1.73 m²	Mild decrease
Na	139 mmol/L	138-145 mmol/L	Normal
K	3.5 mmol/L	3.5-4.8 mmol/L	Normal
Cl	98 mmol/L	96-108 mmol/L	Normal
Ca	8.8 mg/dL	8.8-10.1 mg/dL	Normal
P	3.0 mg/dL	2.7-4.6 mg/dL	Normal
Glucose	101 mg/dL	73-109 mg/dL	Normal
HbA1c	5.8%	4.9-6.0%	Normal
FT3	2.27 pg/mL	2.13-4.07 pg/mL	Normal
FT4	1.47 pg/mL	0.70-1.48 ng/dL	Normal
TSH	3.74 μU/mL	0.35-4.07 μU/mL	Normal
Cortisol	17.47 μg/dL	6.24-18 μg/dL	Normal
ACTH	36.3 pg/mL	7.2-63.3 pg/mL	Normal
KL-6	163 U/mL	<500 U/mL	Normal

**Figure 2:**
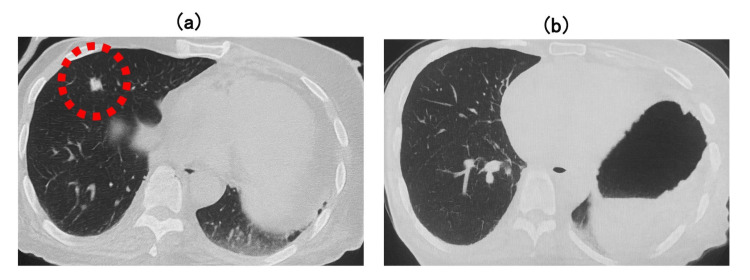
Chest CT images. (a) Computed tomography (CT) before pembrolizumab therapy showing a metastatic pulmonary lesion (red dotted circles). (b) CT scan obtained at the time of the initial syncopal episode showed no evidence of new lesions or progression of pulmonary metastases.

**Figure 3:**
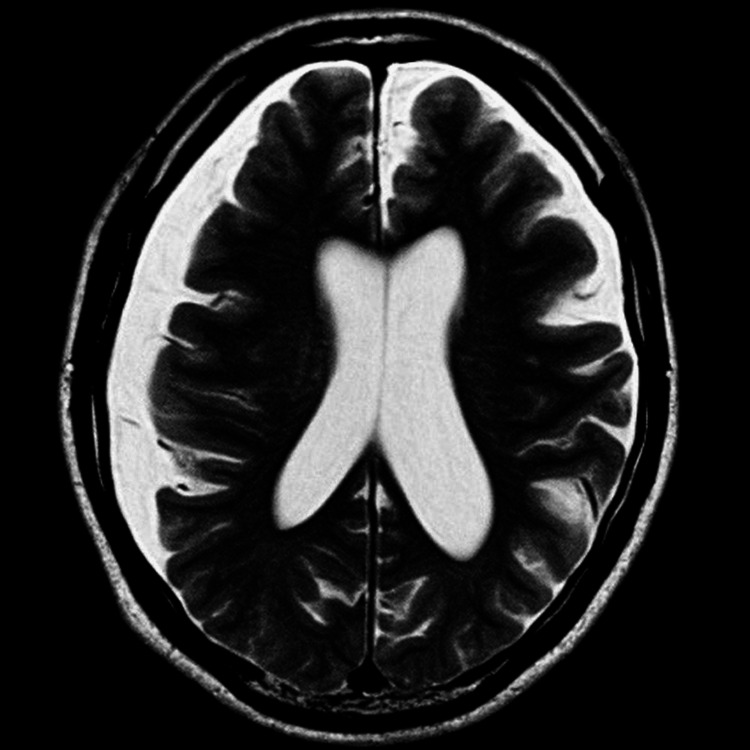
Brain MRI after the first syncopal episode. Brain magnetic resonance imaging (MRI) was performed shortly after the first syncopal episode. No evidence of acute pathology or tumor was found. Preexisting subdural effusion was noted, but no changes were observed compared to imaging performed two years prior to laryngeal cancer surgery.

Computed tomography (CT) demonstrated no evidence of new lesions or disease progression in the throat and neck. It also showed that the pulmonary metastatic lesions remained stable compared to prior imaging taken at the start of pembrolizumab treatment (Figures 2a, 2b). Magnetic resonance imaging (MRI) of the brain revealed only a preexisting right-sided subdural effusion with no evidence of acute pathology or tumor (Figure 3).

The patient was then admitted for observation. No further syncopal episodes occurred during hospitalization, and the patient was discharged once his general condition stabilized. However, on the day of discharge, the patient became unresponsive while returning home in a car driven by a family member. According to the patient’s family, the episode lasted for several minutes. Upon arrival of the emergency personnel, the patient had already regained consciousness, and his vital signs were stable. He was subsequently transported back to the hospital and readmitted for evaluation of recurrent syncope.

During the second hospitalization, the patient underwent further evaluation in the neurology and cardiology departments. Neurological examinations revealed no motor or sensory abnormalities, meningeal signs, tremors, or positive finger-to-nose test results. Electroencephalography revealed no epileptiform discharges. Carotid ultrasonography demonstrated only mild plaque formation in the right internal carotid artery, with no evidence of significant stenosis.

According to the Department of Cardiology, transthoracic echocardiography revealed good left ventricular systolic function, no regional wall motion abnormalities, and no evidence of significant valvular disease. A 24 h Holter monitor showed no arrhythmias, ST changes, or QT prolongation. Finally, a head-up tilt (HUT) test was performed to evaluate autonomic nervous system regulation and a positive result was obtained, with hypotension and bradycardia representing the patient’s symptoms. Vasovagal syncope (VVS) was diagnosed based on the clinical presentation and exclusion of other etiologies.

The patient was managed with behavioral modifications and pharmacological therapy. No further episodes occurred during the second hospitalization, and the patient was discharged in stable condition. Pembrolizumab monotherapy, which had been temporarily withheld during evaluation, was resumed because the syncope episodes were likely unrelated to pembrolizumab directly, and because discontinuing therapy was considered to carry a risk of disease progression. However, several minutes after the 30th pembrolizumab infusion, the patient experienced another brief episode of syncope at the front entrance of the hospital.

**Figure 4:**
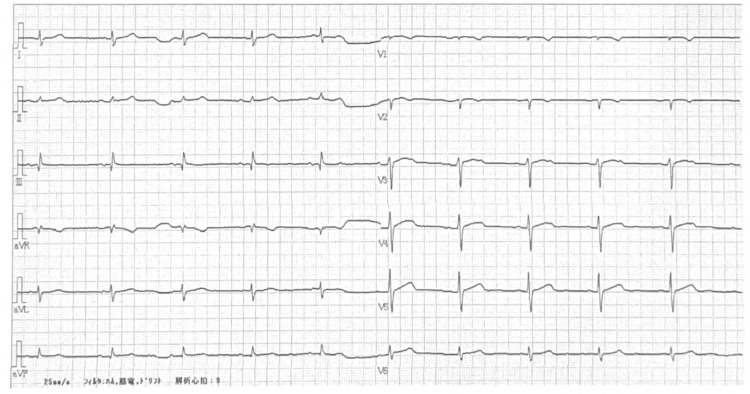
Electrocardiogram (ECG) at the third syncopal episode ECG performed after the third syncopal episode, which occurred shortly after the 30th pembrolizumab infusion. Sinus bradycardia (40 beats/min) was observed without any abnormalities.

At the time of medical staff evaluation, the episode had already resolved. His vital signs were as follows: heart rate 40 beats/min; blood pressure 58/38 mmHg; and SpO_2_ 98% on room air. ECG revealed a sinus rhythm with no newly observed abnormalities (Figure 4). He was monitored for several hours, during which his vital signs remained stable, and no recurrence of symptoms was noted. Given its consistency with prior VVS events, and based on both clinical judgment and patient preference, he was discharged without hospitalization. The subsequent 31st pembrolizumab infusion was completed without complications, and regular administration is being continued.

## Discussion

ICIs such as pembrolizumab have revolutionized the treatment of recurrent or metastatic head and neck cancers. However, their use is associated with a broad spectrum of irAEs, including endocrinopathies, neurological complications, and cardiovascular manifestations [6,7]. Reportedly, 38-48% of patients experience irAEs, with hypothyroidism, dermatitis, and hepatitis being the most frequent. Syncope is defined as a transient loss of consciousness due to transient global cerebral hypoperfusion. Syncope may result from various irAEs, such as adrenal insufficiency, hypophysitis, thyroid dysfunction, or infusion reactions. Therefore, syncope in patients undergoing ICI therapy requires careful diagnostic evaluation to determine immune versus non-immune causes.

In the present case, syncope occurred repeatedly in temporal proximity to pembrolizumab administration, prompting a thorough diagnostic workup to evaluate potential irAEs. A broad range of irAEs was considered in the differential diagnosis, including encephalitis, meningitis, hypophysitis, adrenal insufficiency, and thyroid dysfunction [6,7]. The patient had previously received 28 uneventful cycles of pembrolizumab. Infusion reactions usually occur after the first few doses of pembrolizumab. Late-onset cases, such as reactions following the 41st infusion, have also been reported [8]. However, in this case, infusion reactions were considered unlikely, given the absence of fever, rash, nausea, or wheezing during or immediately after drug administration. Laboratory studies confirmed stable endocrine function. The patient had previously undergone total thyroidectomy, but his thyroid hormone levels were well-controlled with replacement therapy. Brain MRI revealed no evidence of inflammation, acute lesions, or metastatic brain tumors. Neurological examination results, including those of electroencephalography, were unremarkable. Furthermore, repeated episodes of syncope resolved spontaneously without any postictal confusion or neurological deficits, making stroke, encephalitis, or meningitis less likely. The subdural effusion observed on MRI was unrelated to this episode and was diagnosed in the neurology department.

Cardiac causes were excluded based on normal transthoracic echocardiography and Holter monitoring. Collectively, these findings supported the exclusion of life-threatening irAEs and other major etiologies of syncope. VVS is the most common cause of transient loss of consciousness and is typically triggered by autonomic reflex responses to emotional or physical stimuli, such as pain, fear, prolonged standing, or micturition. The diagnosis of VVS is usually clinical and based on the characteristic symptoms and exclusion of other etiologies.

Generally, syncope is classified into the following three categories based on its underlying mechanism: reflex, orthostatic hypotension, and cardiovascular, regardless of whether the patient has cancer or not [9]. In this case, no cardiovascular diseases, such as arrhythmia, were detected on the electrocardiogram and echocardiogram, and cardiac syncope was ruled out.

Carotid sinus syncope, a type of reflex syncope, is generally caused by mechanical manipulation of the carotid sinus, such as strangulation; however, it can also be caused by physical compression due to a neck tumor [10]. However, in this case, CT did not reveal any lesions in the larynx or cervical lymph nodes, which are the primary sites, and carotid sinus syncope was ruled out.

In the present case, a positive HUT test, which reproduced the patient’s symptoms, along with hypotension and bradycardia, was consistent with a vasovagal reflex mechanism, confirming the diagnosis of VVS. A direct association between pembrolizumab use and the onset of VVS has not been clearly established in the existing literature. A previous report described a patient with bladder cancer who experienced syncope during pembrolizumab therapy; however, the episode was ultimately attributed to brain metastasis rather than irAEs [11].

In the present case, although a causal relationship between pembrolizumab and syncope could not be definitively ruled out, the absence of objective findings suggesting side effects of pembrolizumab, including irAEs, and a favorable tumor response supported the decision to continue therapy. Treatment for VVS typically begins with patient education and non-pharmacological approaches, including increased salt and fluid intake, compression garments, and physical counter-maneuvers [12]. Therefore, pharmacological intervention should be considered when these methods are inadequate. Midodrine, an alpha-adrenergic agonist, has shown promise in reducing the recurrence [13,14].

However, despite these measures, recurrence occurred during the continued pembrolizumab therapy. Pacemaker implantation can be selected in cases of refractory vasovagal syncope with significant cardioinhibitory responses induced during HUT testing [15]. The patient in this report is also a candidate for pacing, but there are reports stating that it is not very effective; therefore, pacemakers for refractory vasovagal syncope remain a topic of debate [16,17].

## Conclusions

In patients undergoing ICI therapy, syncope necessitates a comprehensive evaluation to distinguish irAEs from other etiologies. This case illustrates that VVS, although not typically categorized as an irAE, can occur in temporal proximity to pembrolizumab administration. Following a thorough multidisciplinary workup that excluded cardiac, neurological, and endocrine abnormalities, VVS was diagnosed. Given the absence of findings suggestive of irAEs and the favorable oncologic response, pembrolizumab therapy was resumed. This case underscores the importance of considering alternative etiologies such as VVS and supports the continuation of ICI treatment when a causal relationship is not established.

## References

[1] Spain L, Diem S, Larkin J (2016). Management of toxicities of immune checkpoint inhibitors. Cancer Treat Rev.

[2] Martin-Liberal J, de Olza MO, Hierro C, Gros A, Rodon J, Tabernero J (2017). The expanding role of immunotherapy. Cancer Treat Rev.

[3] Zimmer L, Goldinger SM, Hofmann L (2016). Neurological, respiratory, musculoskeletal, cardiac and ocular side-effects of anti-PD-1 therapy. Eur J Cancer.

[4] Jeanmonod R, Sahni D, Silberman M (2023). Vasovagal episode. StatPearls [Internet].

[5] Goyal P, Maurer MS (2016). Syncope in older adults. J Geriatr Cardiol.

[6] Jo W, Won T, Daoud A, Čiháková D (2024). Immune checkpoint inhibitors associated cardiovascular immune-related adverse events. Front Immunol.

[7] Gao L, Li X, Guo Z, Tang L, Peng J, Liu B (2022). Immune checkpoint inhibitor-induced myocarditis with myasthenia gravis overlap syndrome: a case report and literature review. Medicine (Baltimore).

[8] Nakamura T, Imai R, Nishimura N (2022). A case of nonsmall-cell lung cancer with anaphylaxis after 41 courses of pembrolizumab along with adrenal insufficiency as an immune-related adverse event. Case Rep Oncol.

[9] Moya A, Sutton R, Ammirati F (2009). Guidelines for the diagnosis and management of syncope (version 2009): the task force for the diagnosis and management of syncope of the European Society of Cardiology (ESC). Eur Heart J.

[10] Muntz HR, Smith PG (1983). Carotid sinus hypersensitivity: a cause of syncope in patients with tumors of the head and neck. Laryngoscope.

[11] Hatano T, Matsu-Ura T, Mori KI, Inaba H, Endo K, Tamari M, Egawa S (2019). Hyperprogression after pembrolizumab treatment in two patients with metastatic urothelial carcinoma. Jpn J Clin Oncol.

[12] Ballantyne BA, Letourneau-Shesaf S, Raj SR (2021). Management of vasovagal syncope. Auton Neurosci.

[13] Etemadi AQ, Sunthorn H (2014). Vasovagal syncope: diagnosis and management. [Article in French]. Rev Med Suisse.

[14] Lei LY, Raj SR, Sheldon RS (2022). Midodrine for the prevention of vasovagal syncope: a systematic review and meta-analysis. Europace.

[15] Morillo CA, Brignole M (2022). Pacing for vasovagal syncope: tips for use in practice. Auton Neurosci.

[16] Sumiyoshi M (2014). Role of permanent cardiac pacing for vasovagal syncope. J Arrhythm.

[17] Connolly SJ, Sheldon R, Roberts RS, Gent M (1999). The North American Vasovagal Pacemaker Study (VPS): a randomized trial of permanent cardiac pacing for the prevention of vasovagal syncope. J Am Coll Cardiol.

